# Mesenchymal Stem Cell-Derived Extracellular Vesicles Promote Angiogenesis: Potencial Clinical Application

**DOI:** 10.3389/fphys.2016.00024

**Published:** 2016-02-09

**Authors:** Consuelo Merino-González, Felipe A. Zuñiga, Carlos Escudero, Valeska Ormazabal, Camila Reyes, Estefanía Nova-Lamperti, Carlos Salomón, Claudio Aguayo

**Affiliations:** ^1^Department of Clinical Biochemistry and Immunology, Faculty of Pharmacy, University of ConcepciónConcepción, Chile; ^2^Vascular Physiology Laboratory, Group of Investigation in Tumor Angiogenesis (GIANT), Department of Basic Sciences, Universidad del Bío-BíoChillán, Chile; ^3^Group of Research and Innovation in Vascular Health (GRIVAS Health)Chillán, Chile; ^4^Department of Physiopathology, Faculty of Biological Sciences, University of ConcepciónConcepción, Chile; ^5^MRC Centre for Transplantation, King's College LondonLondon, UK; ^6^Exosome Biology Laboratory, Centre for Clinical Diagnostics, University of Queensland Centre for Clinical Research, Royal Brisbane and Women's Hospital, The University of QueenslandBrisbane, QLD, Australia

**Keywords:** mesenchymal stem cell, extracellular vesicles, angiogenesis

## Abstract

Mesenchymal stem cells (MSCs) are adult multipotent stem cells that are able to differentiate into multiple specialized cell types including osteocytes, adipocytes, and chondrocytes. MSCs exert different functions in the body and have recently been predicted to have a major clinical/therapeutic potential. However, the mechanisms of self-renewal and tissue regeneration are not completely understood. It has been shown that the biological effect depends mainly on its paracrine action. Furthermore, it has been reported that the secretion of soluble factors and the release of extracellular vesicles, such as exosomes, could mediate the cellular communication to induce cell-differentiation/self-renewal. This review provides an overview of MSC-derived exosomes in promoting angiogenicity and of the clinical relevance in a therapeutic approach.

## Introduction

Stem cells are defined by their ability to self-renew through replication, resulting in two identical stem cells, and to differentiate into more specialized cells under appropriate conditions (Singer and Caplan, 2011; Mimeault and Batra, 2012). Depending on their origin, they can be classified as embryonic or postnatal/adult stem cells.

Adult stem cells have generated great expectations in the context of regenerative medicine, especially mesenchymal stem cells (MSCs), due to their multi-lineage differentiation potential and their straightforward *in vitro* expansion (Zomer et al., 2015). In particular, MSC transplantation has been suggested as a new promising therapeutic approach for heart, kidney, lung, and liver diseases. Recent studies have however suggested that the beneficial effect of MSCs in cells of injured tissues is not attributed to their differentiation, but rather to their paracrine signaling actions (Caplan and Dennis, 2006).

It has recently been demonstrated that extracellular vesicles or microvesicles (MVs) released from cells are involved in tissue regeneration, and therefore may contribute to the paracrine action of MSCs (Deregibus et al., 2007; Camussi et al., 2010; Mathivanan et al., 2010).

This minireview aims to provide an overview of the role of MSC-derived exosomes in promoting angiogenicity and their therapeutic properties.

## Adult/postnatal stem cells

The bone marrow (BM) is the most extensively investigated source of adult stem cells. The hematopoietic stem cells (HSCs) and the MSCs (or stromal cells) are responsible for the production of blood cells and they are currently the only cell type routinely used for treating patients with hematologic and non-hematologic malignancies (Copelan, 2006). Accumulating evidence has revealed that certain adult stem cells; possess a more broad plasticity and differentiation potential, can circulate in peripheral blood and migrate to tissues/organs and contribute to the promotion of tissue repair at injured sites.

## Mesenchymal stem cells (MSCS)

MSCs were identified for the first time in 1974 by Friedenstein et al. (1974). They described a new cell type, isolated from the bone marrow, with plastic adherent properties and colony forming unit-fibroblasts (CFU-f) capability. Due to their capacity to differentiate into mesenchymal cells such as osteoblasts, adipocytes, and chondroblasts they were denominated MSCs (Caplan, 1991). Recently, the Society for Cellular Therapy proposed the minimum criteria to define MSCs. MSCs: (a) should exhibits plastic adherence (b) possess specific set of cell surface markers, i.e., CD73, CD90, CD105, and (c) ability to differentiate *in vitro* into adipocytes, chondrocytes, and osteoblasts (Dominici et al., 2006).

**Figure 1:**
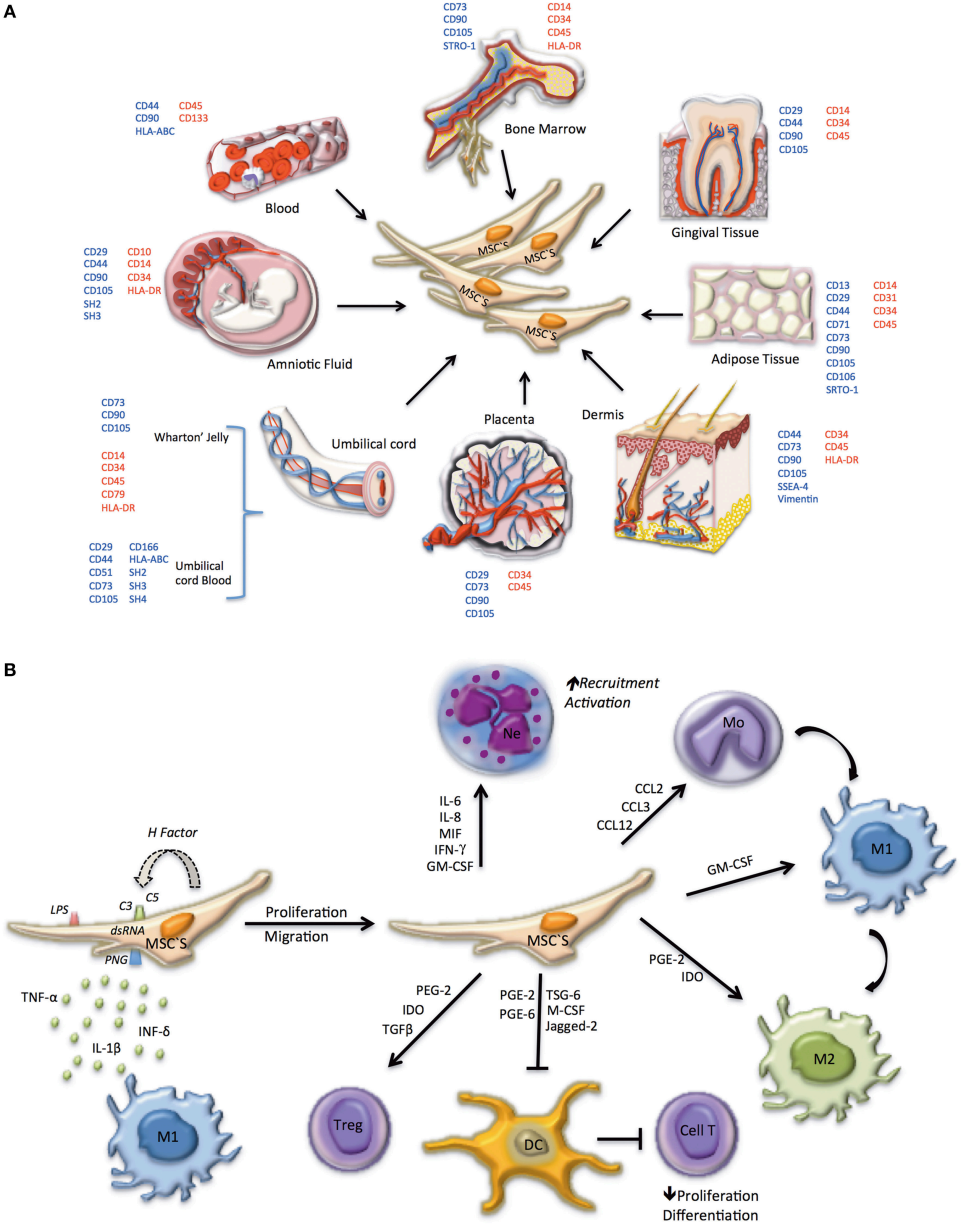
**Phenotype, tissue origin, and immune system regulation of MSC**. **(A)** In embryonic tissues, MSCs can be identified in the amniotic fluid, the wharton's jelly from the umbilical cord, the umbilical cord blood, and in the placenta. In adults MSCs are present in the bone marrow and can migrate to peripheral blood, propagating to several tissues including gingival tissue, adipose tissue, and dermis. Surface markers used to identify MSCs in different locations are indicated; positively expressed markers are shown in blue, negative markers are shown in red. **(B)** MSCs can be differentiated depending on the microenvironment. Initially, MSCs are induced to proliferate and migrate by: molecular patterns associated with pathogens (PAMPs) and LPS (TLR4), dsRNA (TLR3) or PGN (TLR2), activating Toll-like receptors, present on the cell surface of MSCs; secretion of pro-inflammatory cytokines (TNF-α, IFN-γ, IL-1β) by macrophages M1; activation of the complement system (C3, C5 convertase). Factors expressed and secreted by MSCs, such as factor H, regulate the complement system, and prevent cell lysis. If MSC activation occurs early in infection a pro-inflammatory phenotype prevails, promoting the recruitment and activation of neutrophils and monocytes, the latter differentiating to pro-inflammatory macrophages (M1). In advanced stages, MSC take on an anti-inflammatory phenotype, promoting anti-inflammatory macrophage differentiation (M2), tolerogenic dendritic cells (DC) and regulatory T lymphocyte proliferation.

MSCs have been suggested as promising candidates for a variety of therapeutic applications, such as treatment of immune disorders including; systemic lupus erythematosus, bone and cartilage regeneration, neurological diseases, hepatic injury, acute renal failure, and myocardial infarction (Yan et al., 2009; Cao et al., 2010; Xin et al., 2012; Laroni et al., 2013; Wang et al., 2013). MSCs reside in diverse host tissues and organs, such as circulating blood, adult and fetal BM, spleen, amniotic fluid, cartilage, muscle tendons, placenta, adipose tissues, fetal tissues, periosteum, synovial fluid, thymus, trabecular bone, dermis, dental pulp, and lung (Alviano et al., 2007; Battula et al., 2007; Parolini et al., 2008; Mitrano et al., 2010; Salvolini et al., 2010; Figure 1A).

Although several regenerative properties have been ascribed to MSCs, their mechanisms of actions are only partially understood. MSCs synthesize and secrete bioactive factors that modulate the action of adjacent cells. It has been shown that MSCs can have paracrine functions. For example the molecules secreted by MSCs may act as immunomodulators (Carceller et al., 2015), angiogenic factors, (Kinnaird et al., 2004b), anti-apoptotic factors (Khubutiya et al., 2014; Yang et al., 2015) antioxidants molecules (Yang et al., 2015), and/or cellular chemotaxis-inducers (Walter et al., 2015). In addition, MSCs directly or via paracrine action induce fibroblast proliferation, migration, and reduced tissue damage (Liang et al., 2014; Li et al., 2015). Furthermore, these cells exert anti-immflamatory properties, which include the regulation of the innate and the adaptive immune responses (English, 2013; Molina et al., 2015; Figure 1B). Despite the fact that MSCs can modulate molecular and cellular responses directly via cell-cell contact (i.e., cellular differentiation), most studies including ours (Aguilera et al., 2014) indicate that their paracrine effects seems to be also important in relation to tissue repair.

## Extracellular vesicles and exosomes

**Figure 2:**
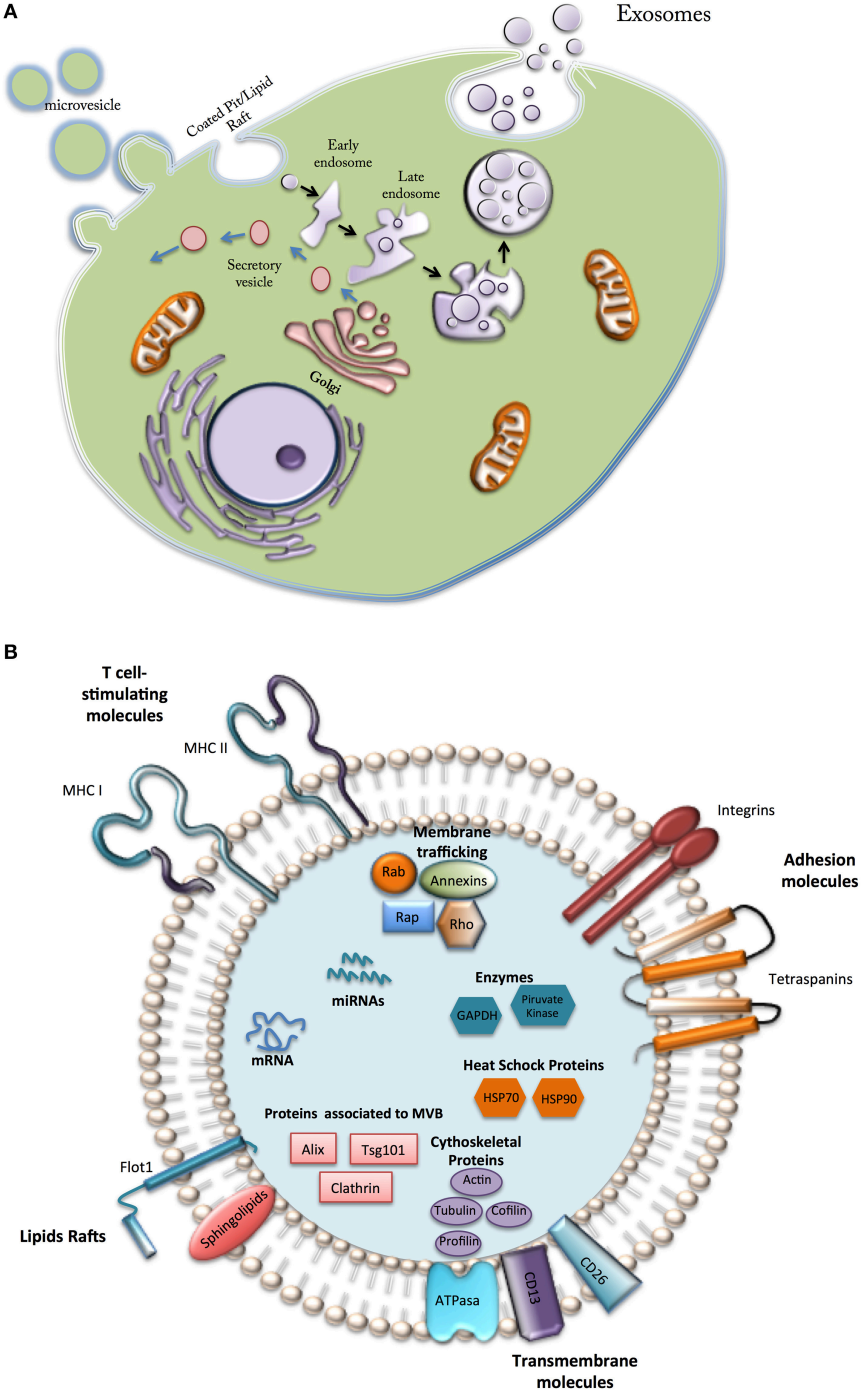
**Types and composition of vesicles secreted by Mesenchymal cells**. **(A)** Microvesicle formed directly from the plasma membrane; secretory vesicles, formed in internal compartments; exosomes, generated from intraluminal vesicles from early endosomes. **(B)** Vesicles from MSCs contain a variety of bioactive components (see text).

Cell secretes a wide range of extracellular vesicles (EVs) of different size, morphology, content and function that interact with target cells and modify their phenotype and function (Colombo et al., 2014; Figure 2A). EVs can be classified according their size, origin, and isolation methods, in to three main classes: (i) Microvesicles or shedding vesicles: size between 50 and 1000 nm, budding from the plasma membrane, isolated by differential centrifugation 10,000–100,000 g, and enriched in CD40; (ii) Apoptotic bodies: size between 800 and 5000 nm, derived from fragments of dying cells, isolated by differential centrifugation 1500–100,000 g, and enriched in histones and DNA; and (iii) Exosomes: which are small (~30–120 nm) membrane vesicles from endocytic origin (therefore, they are enriched in late endosomal membrane markers, including Tsg101, CD63, CD9, and CD81) and formed through the inward budding of multivesicular bodies (MVBs) that traffic and transfect molecules into target cells (Figure 2A).

Although little is known about the mechanism of packaging, exosomes contain a diverse array of signaling molecules (e.g., cell adhesion molecules, growth factor receptors, annexins, Heat-shock proteins). Recent reports have recognized exosomes as cell-derived specific “couriers,” carrying signals and relocating packages of information to modify the biology of target cells (Kastelowitz and Yin, 2014). Exosomes are actively released by a wide range of cells into the local and systemic circulation, playing roles in both physiological and pathological conditions (Colombo et al., 2014).

Progenitor cells release exosomes, which are cardioprotective in ischemia/reperfusion injury (Lai et al., 2010) and can stimulate endothelial cell migration (Vrijsen et al., 2010), cell proliferation (Zhu et al., 2012), tissue vascularization and angiogenesis (Cantaluppi et al., 2012). Exosomes-derived from MSCs promote allograft survival and induce donor-specific allograft tolerance (Plock et al., 2015). Also, exosomes derived from placental MSCs and trophoblast cells promote endothelial cell migration, endothelial tube formation, and extravillous trophoblast migration, respectively (see details in Salomon et al., 2013).

Exosomes may influence the behavior of recipient cells by several different mechanisms, they may act as signaling complexes by direct stimulation of target cells (Kastelowitz and Yin, 2014). Exosomes may interact with target cells by specific receptor ligand interactions and transfer receptors and biological active molecules to these target cells following internalization (Colombo et al., 2014). Additionally exosomes could play a role in the exchange of genetic material between cells. Exosomes are suggested as central mediators of intercellular communication by transferring proteins, mRNAs and miRNAs to adjacent cells leading to coordinative function in organisms. Thus, the microenvironment affecting the releases of exosomes, is critical in influencing the behavior of recipient cells (Valadi et al., 2007; Yuan et al., 2009; Wang et al., 2014).

## Extracellular vesicles and MSC

As described in other cells, vesicles from MSCs contain various exosomal and trophic factors, including growth factors and cytokines (Majore et al., 2011; Patel et al., 2013). They also contain lipids, protein, mRNAs, precursor microRNAs (pre-miRNAs), microRNAs (miRNAs), and transfer RNA (tRNA; Lakkaraju and Rodriguez-Boulan, 2008; Vlassov et al., 2012). Lai et al. (2012), identified 857 proteins inside exosomes released by MSCs-derived embryonic stem cell lines (Lai et al., 2012). In another study, Kim et al. (2011), characterized the protein composition of bone marrow MSC-derived microvesicles (MVs), identifying 730 proteins, including several self-renewal and differentiation mediators. They showed that MSCs vesicles contain platelet-derived growth factor receptor beta (PDGFRB), epidermal growth factor receptor (EGFR), and urokinase-type plasminogen activator receptor (uPAR/PLAUR), which are key factors for promoting cellular changes. The vesicles also contain molecules from the RAS-MAPK, RHO, and CDC42 signaling pathways, suggesting a possible role for these vesicles in tissue repair (Kim et al., 2011; Lai et al., 2015). Other studies found that extracellular vesicles from porcine adipose tissue-derived MSCs contain multiple transcription factors targeting the expression of regulatory genes involved in stem cell survival and function (Dariolli et al., 2013). Other transcription factors linked with development and function of stem cells such as POU class 3 homeobox-1 and POU3F1 (TST-1, OCT6; Wu et al., 2010), along with Jumonji, AT Rich Interactive Domain-2 (JARID2), a transcriptional repressor that plays an essential role in stem cell self-renewal (Hunkapiller et al., 2012), had also been found in extracellular vesicles.

Furthermore, extracellular vesicles also contain mRNAs that regulate apoptosis via the p53 pathway, such as mRNA for Mdm4 p53 binding protein homolog (MDM4; Li and Lozano, 2013) and Paternally Expressed Gene-3 protein (PEG3; Da Silva Meirelles et al., 2009). In addition, multiple miRNAs present in adult MSC-derived exosomes can regulate cell cycle progression and proliferation (miR-191, miR-222, miR-21, let-7a), modulate angiogenesis (miR-222, miR-21, let-7f) and induce endothelial cell differentiation (miR-6087; Yoo et al., 2011). Furthermore, MSCs exosomes are highly enriched in tRNAs, and recent findings showed that tRNA pools from exosomes derived from proliferating cells and differentiating cells were different from each other (Baglio et al., 2015).

Despite growing number of report related with exosomes biology, the entire contents of exosomes are still far from being completely characterized; therefore further studies are required to elucidate the biological function of exosomes in orchestrating tissue repair (Figure 2B).

## Exosomes and angiogenesis

Angiogenesis refers to the formation of new capillaries from existing blood vessels mediated by a complex multistep process of cellular events (Adams and Alitalo, 2007; Bazigou and Makinen, 2013). Several studies focused on identifying angiogenesis stimulators have described: (1) soluble growth factors such as Fibroblast Growth Factor (FGF) and Vascular Endothelial Growth Factor (VEGF), both associated with endothelial cell growth and differentiation (Hoeben et al., 2004); (2) inhibiting factors for proliferation and stimulating differentiation of endothelial cell such as angiogenin (Sovak et al., 1999); and (3) extracellular cytokines, such as angiostatin and endostatin (Shih and Lindley, 2006).

Proteomic analysis showed that exosomes derived from MSCs contain growth factors such as VEGF, TGFB1, and interleukin-8 (IL-8), which have been shown to contribute in their pro-angiogenic activity (Coultas et al., 2005; Olsson et al., 2006). In addition, is known that MSC-derived extracellular vesicles are also rich in transcription factors involved in pro-angiogenic pathways, such as Hepatocyte Growth Factor (HGF) that stimulates proliferation and migration of endothelial and vascular smooth muscle cells (Morishita et al., 1999; Chade and Stewart, 2013; Tan et al., 2014). HES Family BHLH Transcription Factor 1 (HES1) is a critical downstream effector of the Notch signaling pathway that regulates vascular remodeling and arterial fate of endothelial cells (Kitagawa et al., 2013). Similarly, Human T-cell factor 4 (TCF4) is a key downstream effector of Wnt signaling, a canonical pathway that plays a central role in vascular development (Maruotti et al., 2013) and in determining and maintaining the phenotype and functional properties of human stem cells (Lu et al., 2012). Therefore, intercellular transmission of EVs containing HGF, HES1, and TCF4 may have both pro-angiogenic and pro-survival effects.

In addition, recent studies have reported that exosomes and extracellular vesicles could carry Wnt on their surface to induce Wnt signaling activity in target cells (Gross et al., 2012; Reis and Liebner, 2013; Pate et al., 2014). Results from Zhang et al. (2015) demonstrated that exosomes derived from human umbilical cord mesenchymal stem cells (hucMSC-Ex) enhance angiogenesis in the repair of skin second-degree burn injury. Additionally, they found that knockdown of Wnt4 in hucMSC-Ex delays tube formation of endothelial cells *in vitro* and the expression of CD31 *in vivo* (Zhang et al., 2015). These results confirm participation of the Wnt-pathway in a pro-angiogenic and tissue repair role, mediated by MSC-derived exosomes.

Extracellular vesicles derived from adipose mesenchymal stem cells (ASC-EV) contain a set of angiogenic factors such as MFG-E8, ANGPTL1, thrombopoietin (Lopatina et al., 2014). Moreover, ASC-EV were found to carry matrix metalloproteinases (MMPs) that play an important role in angiogenesis by facilitating endothelial cell migration and by promoting activation of angiogenic growth factors and other signaling molecules (Lee et al., 2013).

In migrations assay using Human Umbilical Vein Endothelial Cells (HUVECs), the numbers of migrated cells and the tube length in matrigel analysis increased significantly after treatment with conditioned medium harvested from MSCs primary culture (MSC-CM) or MSC-exosomes, compared with the control (Kinnaird et al., 2004a). In addition, *in vitro* experiments from the same study showed that the MSC-derived exosomes significantly promoted myogenesis and angiogenesis compared to MSC-conditioned media (MSC-CM) (Nakamura et al., 2015). However, MSCs-derived exosomes exhibit significantly lower levels of VEGF and IL-6 than MSC-CM. This confirms that the paracrine effects of MSCs are not only attributable to cytokines and growth factors, but also to other factors, including specific exosome factors (Nakamura et al., 2015).

One group of molecules with “paracrine” signaling capacity identified inside exosomes are miRNAs. Exosomes derived from MSCs contain several miRNAs, including miR210, miR126, miR132, and miR21, which have all been shown to play central roles in angiogenesis (Chen et al., 2010). In addition, multiple miRNAs in adult MSC-derived exosomes regulate cell cycle progression and proliferation (miR-191, miR-222, miR-21, let-7a), and promote angiogenesis (miR-222, miR-21, let-7f) and endothelial cell differentiation (miR- 6087; Yoo et al., 2011; Nagpal and Kulshreshtha, 2014). So far, at least 20 miRNAs have been found in human bone-marrow-derived MSCs exosomes. Other miRNAs such as miR-1, miR-133, and miR-206 have been also detected in human bone-marrow-derived MSCs exosomes (Nakamura et al., 2015).

Angiogenesis is only one of multiple effects of exosomes which have been associated with; proliferation and migration of endothelial cells and vascular smooth muscle cells, differentiation into endothelial cells and vascular smooth muscle cells, and formation of endothelial cells from formerly existing vessels, enhanced blood flow restoration, and capillary network formation. Suggesting that Exosomes may be a novel therapeutic approach in the treatment of ischemic diseases.

## Potential clinical use of exosomes derived from MSCs

The paracrine effects of MSCs therapy have been previously reported in a wide range of disease (Galderisi and Giordano, 2014; Rani et al., 2015). For instance, Kang et al. (2015) examined the role of exosomes derived from rat bone marrow MSCs on cardiac functions in a rat model of myocardial infarction (Kang et al., 2015). They observed that exosomes protect cardiomyocytes from ischemic injury both *in vitro* and *in vivo* by acting on hearts and vessels, promoting cardiac regeneration mediated by neovascularization and anti-vascular remodeling (Huang et al., 2015). This study also showed that exosomes secreted by MSCs were able to reduce myocardial ischemia/reperfusion injury (Huang et al., 2015). Moreover, exosomes from MSCs overexpressing CXCR4 showed better efficiency for reducing left ventricular remodeling and promoting restoration of heart function (Kang et al., 2015).

Other studies have reported that intact exosomes secreted by MSCs reduced oxidative stress, increased ATP and NADH production, controlled inflammatory activities and activated the PI3K/Akt pathway, which in turn leads to protective influences on cardiomyocytes as well as survival and retention of left ventricular function after ischemia-reperfusion injury (Khan et al., 2013; Huang et al., 2015). Similarly, MSC-derived EV reduced the infarct size in a pig model of ischemia/reperfusion injury (Timmers et al., 2008). In another study, *in vivo* analysis of a mouse model of muscle injury showed that injection of MSC-exosomes accelerated muscular regeneration, enhanced angiogenesis, and reduced fibrosis (Nakamura et al., 2015).

In the lung, MSCs-derived exosomes suppress the activation of the hypoxic signal pathway, activated alveolar macrophages, mediated by down-regulation of proliferative miR-17 (Lee et al., 2012). Moreover, MSC-derived exosomes might also disturb STAT3-miR-204-STAT3 feedback to ameliorate vascular remodeling (Huang et al., 2015). The findings above suggest that MSC-derived exosomes induce lung protection via preventing early pulmonary inflammation and vascular remodeling.

In addition, in the kidney, MSC-derived extracellular vesicles exert pro-survival effect on renal cells by inducing the expression of anti-apoptotic genes and down-regulating the expression of pro-apoptotic genes (Bruno et al., 2012; Zhou et al., 2013). Furthermore, other studies suggested that exosomes from MSCs could protects against liver fibrosis (Li et al., 2012), and acute kidney injury (Bruno et al., 2012; He et al., 2012), and enhance cutaneous wound closure. Therefore, is possible that MSCs-exosomes promote cell proliferation and subsequently, injury repair, and wound healing.

Nevertheless in cancer, exosomes released and/or delivered into tumor microenvironment can modulate epithelial to mesenchymal transition, cancer stemess, angiogenesis, and metastasis (Higginbotham et al., 2011; Alderton, 2012; Lee et al., 2013; Yu et al., 2015). Recent studies have shown that chemotherapy enhanced the secretion of exosomes in tumor cells, leading to the transfer of chemoresistance related miRNAs and mRNAs to neighboring cells to alter their sensitivity to chemotherapy, suggesting a critical role of exosomes in the cellular response to chemotherapy (Yang et al., 2011; Kahlert and Kalluri, 2013).

Thus, several studies have reported that MSC-derived exosomes have functions similar to those of MSCs, such as repairing tissue damage, suppressing inflammatory responses, and modulating the immune system. This paracrine action of MSCs has changed the perspective of their use in regenerative medicine. The use of MSCs may attenuate many of the safety concerns related to the use of living cells.

## Concluding remarks

Cell-based therapies have been used with high efficacy in clinical trials for stroke, neurological disorders and other diseases. However, several studies have shown multiple benefits of using exosomes rather than cells. For example, MSC-derived MVs could reduce potential risks associated with cellular therapies, including ectopic tissue formation, infusion toxicities due to cells lodging and cellular rejection or unwanted engraftment. It is known that exosomes carry and transfer their cargo to parenchymal cells, where they mediate cellular plasticity and functional recovery in pathological conditions via paracrine signaling. However, the detailed mechanisms underlying the benefits of exosomes in MSCs transplantation in these diseases requires further investigations. Thus, exosomes derived from MSCs represents a promising approach for the treatment of human disease.

In conclusion, this report offers a comprehensive analysis of extracellular vesicular cargo that sheds light on the cellular communication. In this review, we highlight that exosomes from stressed MSCs act as rafts to carry supportive proteins, miRNA, lipids and metabolites. Further studies are required to determine which of these components trigger the right molecular mechanisms in the tissue of interest.

## Author contributions

This work was carried out as a full collaboration among all the authors. CA defined the research topic and all co-wrote the manuscript and approved the final version of the manuscript.

### Conflict of interest statement

The authors declare that the research was conducted in the absence of any commercial or financial relationships that could be construed as a potential conflict of interest.
